# Rheumatic mitral valve disease: Comparative outcomes of repair versus replacement

**DOI:** 10.21542/gcsp.2025.60

**Published:** 2025-12-31

**Authors:** Sofia Araujo, Hugo Silva, Renato Filho, Natalia Alves, Arthur de Lima, João Aires, Fernanda Chaves

**Affiliations:** Universidade de Pernambuco, Faculdade de Ciências Médicas de Pernambuco, Rua Arnóbio Marques, 310, Bairro de Santo Amaro, Recife-PE, CEP 50100-130, Brazil

## Abstract

Rheumatic mitral valve disease remains a major cause of cardiovascular morbidity and mortality in developing countries where rheumatic fever is still prevalent. In advanced stages, surgical treatment becomes necessary, with mitral valve repair/valvuloplasty (MVP) and mitral valve replacement (MVR) representing the main strategies. However, evidence comparing their long-term outcomes is heterogeneous and often conflicting. The objective of this study was to map and synthesize the scientific evidence comparing the long-term results of repair versus replacement in rheumatic mitral disease. A systematic review was conducted in accordance with established methodological guidelines. Searches were performed in PubMed, Virtual Health Library, and Cochrane databases. Eligible studies, published between 2016 and April 2025, included adult patients undergoing surgical treatment with follow-up of at least five years. Nine studies met the inclusion criteria, comprising 36,136 patients, with 12,042 undergoing repair and 24,094 undergoing replacements. Repair was associated with lower early mortality (0.96% versus 2.2%), fewer thromboembolic events (114 versus 323), fewer hemorrhagic events (39 versus 252), and better preservation of left ventricular function. However, reoperation rates were higher after repair (7–19%) compared to replacement (<2%). Quality-of-life measures also favored repair, while replacement remained the preferred strategy in cases of extensive calcification, fibrosis, or severe stenosis. In conclusion, repair, when anatomically feasible, provides superior early outcomes and fewer long-term complications. Surgical decisions should be individualized, considering valve anatomy, patient profile, and surgical expertise.

## 1. Introduction

Cardiovascular diseases remain the leading cause of mortality worldwide, with valvular heart disease making an important contribution to this scenario^1^. Among these, rheumatic mitral valve disease remains highly prevalent in low- and middle-income countries such as Brazil, due to the persistence of rheumatic fever^2,4^. As a consequence, chronic inflammation leads to progressive structural changes in the mitral valve, resulting in stenosis, insufficiency or mixed lesions, compromising cardiac function and reducing patients’ quality of life^4,9^.

In the more advanced stages of the disease, surgical intervention is necessary^1,2^. The main approaches are mitral valve repair, which aims to restore the functionality of the native valve, and mitral valve replacement, which completely replaces the valve with a mechanical or biological prosthesis^3,5^. The choice between these techniques depends on various factors, such as the anatomical severity of the lesion, the patient’s clinical profile, the presence of comorbidities, life expectancy and the experience of the surgical team^5^.

Mitral valve repair has advantages such as better preservation of left ventricular function, less need for anticoagulation and lower perioperative mortality^3,6^. However, it is technically more challenging in rheumatic valves, which are often affected by fibrosis and calcification, and may be associated with a higher risk of long-term reoperation^6,8^. On the other hand, valve replacement is more widely used and technically accessible, but involves risks such as thromboembolic events, prosthetic deterioration and the need for continuous anticoagulation, especially with the use of mechanical prostheses^3,6,8^.

Several recent studies have sought to compare the clinical outcomes of these approaches in patients with rheumatic mitral valve disease^1,4,6^. Chen et al. observed differences in survival and reoperation rates between valvuloplasty and valve replacement^1^, while Brescia et al. discussed the importance of surgical choice in view of the possibilities of reintervention^2^. In addition, one study compared mechanical and biological prostheses, highlighting the influence of the prosthetic type on outcomes^3^.

Despite these contributions, the literature still has significant gaps^6–8^. Many studies include multiple etiologies of mitral valve disease (degenerative, functional and rheumatic), which limits the specific interpretation of rheumatic disease^6,7^. Furthermore, the results are heterogeneous and sometimes conflicting: while some studies suggest lower mortality with repair^1,4^, others point to a higher risk of reoperation in this group^8^.

These inconsistencies reinforce the need to systematically map the available evidence that directly compares clinical outcomes between repair and valve replacement exclusively in patients with rheumatic mitral valve disease.

Given this scenario, this systematic review aims to identify, synthesize and analyze the available scientific evidence on the clinical outcomes associated with mitral valve repair compared to mitral valve replacement in patients with rheumatic mitral valve disease. Outcomes such as perioperative mortality, reoperations, thromboembolic events and quality of life will be considered in order to support evidence-based clinical practice and point out gaps that justify future research.

## 2. Methodology

The present study consists of a systematic literature review, conducted in accordance with the recommendations of the Preferred Reporting Items for Systematic Reviews and Meta-Analyses (PRISMA) statement, a tool widely recognized for its effectiveness in the rigorous and transparent synthesis of scientific evidence. Systematic reviews aim to integrate and critically evaluate the findings from primary studies on a well-defined research question, employing a structured methodological protocol to minimize biases and ensure reproducibility^10^.

This approach is particularly relevant when seeking to establish robust conclusions from multiple sources of evidence, as it allows for the identification of patterns, contradictions, or consensus within the literature^10^.

It should be noted that the protocol for this systematic review was not previously registered in a platform such as PROSPERO. This decision was due to the initial exploratory nature of the review, which aimed to first map the feasibility and volume of available literature before committing to a registered protocol. All subsequent methodological steps, however, were rigorously followed to ensure the transparency and reproducibility of the process.

In the included studies, different effect measures were used to quantify clinical outcomes, each appropriate to a specific type of data and study design. The Relative Risk (RR) expresses the ratio between the probability of an outcome in the intervention group and in the comparison group, and is most appropriate in cohort studies reporting cumulative incidence. The Odds Ratio (OR), which represents the ratio between the odds of an event occurring in one group versus another, is typically used in case–control studies or when the outcome is rare. Hazard Ratios (HR), derived from time-to-event analyses (survival analysis), compare the instantaneous risk of an event over time between groups and are suitable for studies with censored follow-up. Subdistribution Hazard Ratios (SHR), used particularly in Fine-Gray competing-risk models, allow estimation of the effect of an exposure on the cumulative incidence of an outcome when competing events (e.g., death before reoperation) may prevent its occurrence. These distinctions are essential for correctly interpreting the findings of long-term surgical outcomes in rheumatic mitral valve disease, where follow-up time, censoring and competing risks are frequent methodological elements.

### 2.1. Searching databases

The study began with the formulation of a guiding question, using the PICO (Population, Intervention, Comparison, Outcomes) methodological strategy. The strategy generated the following question: “What are the long-term clinical outcomes of valve repair compared to valve replacement in patients with rheumatic mitral valve disease?”

In order to clarify the guiding question, searches were carried out in the PUBMED, Cochrane and VHL databases in March and April 2025. Controlled descriptors from the MeSH (Medical Subject Headings) and DeCS (Descriptors in Health Sciences) terminologies were used, associated by boolean operators. The search strategy for each database was carried out taking into account the particularities of each search system.

**Figure 1.**
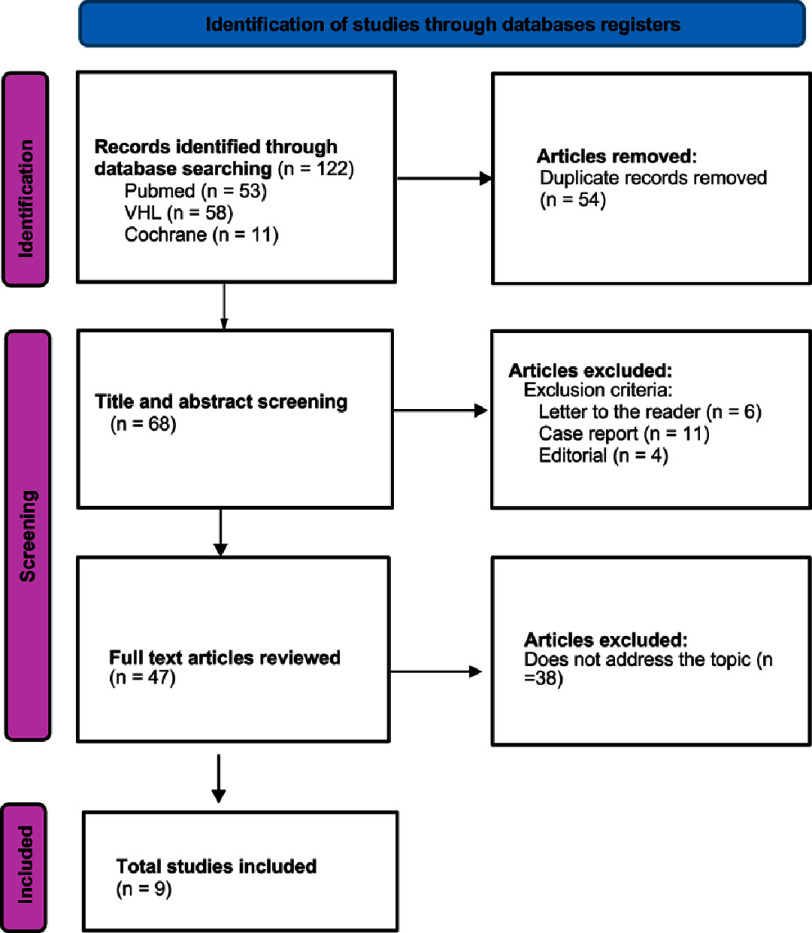
Screening of articles using the systematic review flowchart (PRISMA).

### 2.2. Search strategy used in each database

Pubmed: (“Mitral Valve Repair”[MeSH] OR “Valvuloplasty”[MeSH] OR “mitral valve repair” OR “valvuloplasty”) AND (“Mitral Valve Replacement”[MeSH] OR “Valve Replacement”[MeSH] OR “mitral valve replacement” OR “valve replacement”) AND (“Rheumatic Heart Disease”[MeSH] OR “Rheumatic Mitral Valve Disease” OR “rheumatic heart disease” OR “rheumatic mitral valve disease”) AND (“Clinical Outcomes”[MeSH] OR “mortality” OR “morbidity” OR “complications” OR “survival rate” OR “long-term outcomes” OR “postoperative outcomes”);

BVS: (“Mitral Valve Repair” OR “Valvuloplasty”) AND (“Mitral Valve Replacement” OR “Valve Replacement”) AND (“Rheumatic Heart Disease” OR “Rheumatic Mitral Valve Disease”) AND (“Clinical Outcomes” OR “Mortality” OR “Morbidity” OR “Complications” OR “Survival Rate” OR “Long-Term Outcomes” OR “Postoperative Outcomes”);

Cochrane: (“mitral valve repair” OR valvuloplasty OR “valve reconstruction” OR “valve preservation” OR “valve sparing”) AND (“mitral valve replacement” OR “valve replacement” OR “prosthetic mitral valve” OR “mechanical valve” OR “biological valve” OR “valve prosthesis”) AND (“rheumatic heart disease” OR “rheumatic mitral valve disease” OR “rheumatic valve disease” OR “rheumatic fever” OR “valvular heart disease” OR “chronic rheumatic heart disease”) AND (“clinical outcomes” OR mortality OR morbidity OR complications OR “survival rate” OR “long-term outcomes” OR “postoperative outcomes” OR “hospitalization” OR “reoperation” OR “functional outcomes” OR “quality of life”).

### 2.3. Eligibility and screening criteria

The main inclusion criteria were: articles with free access to the full text, published from 2016 to April 2025, in any language and comparing valve repair and valve replacement operations performed on adult patients with rheumatic mitral valve disease. Long-term clinical outcomes (≥5 years) such as mortality, morbidity and quality of life were considered. Case reports, letters to the reader, editorials and studies that did not directly address the topic proposed during the screening were excluded.

As illustrated in the PRISMA diagram (Figure 1), a total of 122 articles were found in the database search, 53 from PubMed, 58 from the Virtual Health Library (VHL) and 11 from Cochrane. After the initial selection, the articles were screened on the Rayyan platform (free version). The screening was carried out by two independent reviewers (J.P. and R.V.) and disagreements were resolved by consensus, through joint analysis and discussion. Initially, 54 of the 122 articles were excluded due to duplication. After reading the titles and abstracts, 21 articles were excluded because their methodological design was not compatible with the aim of the review, such as letters to the reader (6), case reports (11) and editorials (4). The remaining articles were fully analyzed by the reviewers and 38 were excluded because they did not directly address the comparison between valve replacement and valve repair, because they were studies on non-rheumatic valve diseases, non-surgical interventions or inadequate outcomes (e.g., follow-up < 5 years, lack of quantitative data), leaving 9 articles at the end of the screening, all of them in English.

Data was extracted using a structured spreadsheet in Google Sheets, containing the following variables: authors, periodical/year, objective, type of study, results and level of evidence. A formal risk-of-bias assessment was performed using the Newcastle–Ottawa Scale (NOS) for observational cohort studies and the Cochrane Risk of Bias 2 (RoB 2) tool for clinical trials. Secondary studies (systematic reviews/meta-analyses) were qualitatively appraised but not scored with these instruments. The process was conducted by a single reviewer (S. A.), and the details are in the results section.

## 3. Results

The database search resulted in the identification of 122 articles. After removing 54 duplicates, 68 studies were screened by title and abstract. Of these, 38 were excluded because they were not in line with the proposed theme. Full reading of the remaining 30 articles led to the exclusion of a further 21 because they did not meet the eligibility criteria. In the end, 9 studies were included in this systematic review, as shown in Figure 1 (PRISMA Diagram).

In all, the articles analyzed 36,136 patients. Of this total, 12,042 (33%) underwent mitral valve repair, while 24,094 (67%) underwent valve replacement, either with a mechanical or biological prosthesis. The study populations were mainly concentrated in countries with a high incidence of rheumatic fever, such as China, Taiwan, India and Brazil. All the studies included were published between 2020 and 2024. In terms of methodological design, the following were considered: 2 retrospective cohort studies, 1 comparative study, 1 prospective observational study, 2 clinical trials, 2 systematic reviews and 1 meta-analysis. This can be seen in Table 1, which shows the articles selected with their respective descript.

**Table 1:** Summary of the studies included in the systematic review.

**Study**	**Journal/Year**	**Objective**	**Study type**	**Key results**	**Level of evidence**
**Mitral valve repair versus replacement in patients with rheumatic heart disease** Chen et al.	*Thorac Cardiovasc Surg*/2022	Compare long-term outcomes between MVR and MVP in RHD	Retrospective Cohort	• Hospital mortality similar: Repair 33.4% vs. Replacement 32.5% (HR 1.01; 95% CI: 0.81–1.25) • Replacement had lower reoperation risk (SHR 4.32; 95% CI: 2.02–9.23)	III
**Outcomes of mitral valve repair or replacement in the valve-in-valve era** Brescia et al.	*Thorac Cardiovasc Surg*/2022	Evaluate decision-making between repair vs. replacement and outcomes	Comparative Study	• 5-year survival: Replacement 81% ± 9% vs. Repair 78% ± 10% (*P* = 0.70) • Reoperation: Replacement 3.7% vs. Repair 17% (*P* = 0.014)	III
**Bioprosthetic versus mechanical mitral valve replacement in patients with rheumatic heart disease** Chen et al.	*Thorac Cardiovasc Surg*/2023	Compare long-term outcomes of bioprosthetic vs. mechanical valves in RHD	Retrospective Cohort	• 10-year mortality: Mechanical 45.5% vs. Bioprosthetic 50.6% (HR 1.19; 95% CI: 1.01–1.41; *P* = 0.040) • Reoperation: Mechanical 0.93% vs. Bioprosthetic 8.9% (SHR 4.56; 95% CI: 1.71–12.17)	III
**Outcomes of mitral valve repair compared with replacement for patients with rheumatic heart disease** Fu et al.	*Thorac Cardiovasc Surg*/2021	Compare outcomes of repair vs. replacement using propensity score matching	Prospective Observational	• Early mortality: Repair 0.6% vs. Replacement 3.0% (HR 0.19; 95% CI: 0.05–0.64]; *P* = 0.003) • Fewer valve complications with repair (SHR 0.44; 95% CI: 0.21–0.90; *P* = 0.025)	III
**Short and mid-term effects of modified release technique in rheumatic mitral valve repair** Chong et al.	*J Cardiothorac Surg*/2023	Evaluate outcomes of modified release technique	Clinical Trial	• Shorter CPB time: 105 ± 15 vs. 125 ± 28 min (*P* < 0.01) • Lower transvalvular gradient: 2.7 ± 1.1 vs. 3.8 ± 1.2 mmHg (*P* = 0.01)	III
**Comparative efficacy and safety of mitral valve repair versus mitral valve replacement in rheumatic heart disease** Yasmin et al.	*Curr Probl Cardiol*/2024	Determine efficacy and safety of MVP vs. MVR in RHD	Systematic Review/Meta-analysis	• MVP: Reduced early mortality (RR 0.63; *P* = 0.003), better long-term survival (HR 0.53; *P* = 0.0009) • 12-year survival: 81.0% (MVP) vs. 74.6% (MVR) • Higher reoperation risk with MVP	I
**Outcomes of Mitral Valve Surgery in Patients With Rheumatic Disease: Repair vs. Replacement** Fu et al.	*Front Cardiovasc Med*/2021	Compare perioperative and long-term outcomes of MVP vs. MVR	Systematic Review	• MVP: Higher long-term survival (HR 0.72), lower early mortality (RR 0.62) • Higher reoperation risk (HR 2.60)	III
**Clinical outcomes following surgical mitral valve repair or replacement in patients with rheumatic heart disease** Jiang et al.	*Ann Transl Med*/2021	Evaluate suitability of MVP in RHD, comparing valve types	Meta-analysis	• MVP: Lower early mortality (OR 0.58), late mortality (HR 0.49) • Higher reoperation risk vs. mechanical (HR 2.4), similar to bioprosthetic (HR 0.8)	I
**The effect of rheumatic mitral valve repair using ”standardized 4 steps”** Zhang et al.	*Chinese J Thorac Cardiovasc Surg*/2020	Investigate clinical effect of standardized 4-step repair technique	Clinical Trial	• Perioperative mortality: 2.94% • Significant reduction in LA diameter: 54.3 ± 6.8 to 46.7 ± 5.9 mm (*P* < 0.05) • No significant difference vs. replacement in early outcomes	III

### 3.1. Mortality

Only 66% of the studies included^1,2,4,6,7,9^ reported quantitative data on mortality. In these studies, 648 deaths were recorded among the 23,523 patients evaluated. Of these, 116 occurred in patients who underwent mitral valve repair (0.96%; 95%CI 1.42–2.04%), while 532 were observed among those who underwent valve replacement (2.2%; 95%CI 2.90–3.44%). Most deaths occurred in the early postoperative period. Mitral valve repair was associated with lower mortality in all the studies that reported this outcome, with a relative reduction of up to 70% in early mortality, especially in patients with favorable valve anatomy^4^.

### 3.2. Reoperation

Around 88% of the studies^1–4,6–9^ provided information on the need for reoperations. A total of 429 reoperations were recorded, 364 in the plasty group (3.96%; 95%CI 3.57–4.36%) and 65 in the replacement group (0.244%; 95%CI 0.185–0.303%). Reoperation rates ranged from 7% to 19% for mitral valve repair, indicating less durability of the repair compared to valve replacement, which had rates of less than 2%^4^. Studies using standardized repair techniques^5,6,9^ have reported reoperation rates of less than 6% over five years, suggesting improved repair durability in these cases.

**Table 2:** Risk of bias assessment of the included studies. Observational cohorts were evaluated using the Newcastle–Ottawa Scale (NOS), while clinical trials were assessed using the Cochrane Risk of Bias 2 (RoB 2) tool. Secondary studies (systematic reviews and meta-analyses) were qualitatively appraised but not scored.

**Study**	**Study design**	**Risk-of-bias tool**	**Overall risk of bias**	**Main Concerns**
**Chen et al., 2022**	Retrospective cohort (national administrative database) with 1:1 propensity score matching	NOS	**Moderate**	Observational design; repair performed in a small minority of patients; residual confounding and selection bias likely despite matching; limited echocardiographic detail
**Brescia et al., 2022**	Retrospective comparative study across two eras	NOS	**High**	Marked temporal changes in practice (Era 1 vs Era 2); no propensity matching; unequal follow-up; strong risk of confounding by era and surgeon preference
**Chen et al., 2023**	Retrospective cohort comparing mechanical vs bioprosthetic replacement with propensity score matching	NOS	**Moderate**	Large sample and matching, but reliance on administrative data; incomplete adjustment for clinical and anatomical variables; residual confounding cannot be excluded
**Fu et al., 2021**(cohort)	Prospective observational study with propensity score matching	NOS	**Low**	Prospective design, standardized data collection and robust matching; well-defined outcomes and follow-up; remaining risk mainly from unmeasured confounders
**Chong et al., 2023**	Non-randomised clinical trial comparing modified repair vs replacement	RoB 2	**High**	Allocation not randomised; no description of allocation concealment or blinding; selection of patients for the modified technique; short and variable follow-up
**Zhang et al., 2020**	Retrospective cohort comparing repair vs replacement	NOS	**High**	Strong treatment-selection bias (repaired valves less extensively diseased); lack of propensity matching or multivariable adjustment; incomplete control of confounders

### 3.3. Thromboembolic and hemorrhagic events

Approximately 77% of the studies^1,4,6–9^ reported data on thromboembolic and hemorrhagic events. A total of 437 thromboembolic events were identified, with 114 occurring in the plasty group (1.25%; 95%CI 1.02–1.48%) and 323 in the replacement group (1.41%; 95%CI 1.26–1.56%). In addition, 291 hemorrhagic events were recorded, 39 in the plasty group (0.428%; 95%CI 0.294–0.562%) and 252 in the replacement group (1.10%; 95%CI 0.965–1.235%). The lower incidence of complications in the plasty group was attributed to the absence of the need for continuous oral anticoagulation, which is mandatory when using mechanical prostheses^9^.

### 3.4. Cardiac function and hemodynamic parameters

Of all the studies, 66% presented data on hemodynamic performance^5–7,9^. Among them, some reported^5,7,9^ mean transvalvular gradients of 2.5–2.9 mmHg in the plasty group and 3.5–4.2 mmHg in the replacement group (the individual studies did not report confidence intervals for these means). A mean ejection fraction of 60.7% for plasty and 57.1% for replacement was observed in three studies^4,6,7^; in study 4 specifically, LVEF < 50% occurred in 20 patients in the plasty group (5.17%; 95%CI 4.05–6.29) and 21 in the replacement group (5.43%; 95%CI 4.27–6.58). In addition, a 20-minute shorter cardiopulmonary bypass time in the plasty group was reported by two studies^5,9^. These data suggest better immediate hemodynamic performance after mitral valve repair.

### 3.5. Patient profile and therapeutic indications

Around 55% of the studies^1–3,6,7^ discussed patient profiles and therapeutic indications. Mitral valve repair was most indicated in young patients with predominant mitral regurgitation, anatomy favorable to repair and absence of significant calcifications. Valve replacement was preferred in cases with severe mitral stenosis, extensive fibrosis, a history of percutaneous commissurotomy or the need for simultaneous surgical intervention on other heart valves^6^.

### 3.6. Surgical strategies and current practices

Only 33% of the studies^5,6,9^ reported the use of standardized mitral valve repair techniques, such as multi-stage commissureplasty and the use of autologous pericardium. These studies associated these techniques with good functional results and lower reoperation rates. In addition, they reported shorter hospital stays and reduced need for inotropic support in the postoperative period.

Overall, the data gathered in this review suggests that mitral valve repair can be an excellent alternative for the surgical treatment of rheumatic mitral valve disease, especially in young patients with anatomy favorable to repair. Valve replacement, on the other hand, remains a valid strategy, especially in more complex cases, such as in the presence of significant stenosis or extensive calcifications. Even so, there is a need for more research to confirm these findings and expand knowledge about the clinical outcomes associated with each approach. This will contribute to more assertive surgical decisions, based on evidence and adapted to each patient’s profile.

### 3.7. Risk of bias of included studies

A formal risk-of-bias assessment was performed using the Newcastle–Ottawa Scale (NOS) for observational cohort studies and the Cochrane Risk of Bias 2 (RoB 2) tool for clinical trials. Secondary studies (systematic reviews/meta-analyses) were qualitatively appraised but not scored with these instruments. Overall, risk of bias among the primary studies ranged from low to high (figure 3). The only prospective, propensity-matched cohort^4^ was judged to be at low risk of bias.

Large retrospective database cohorts with propensity score matching^1,3^ were rated as having moderate risk of bias, reflecting residual confounding and limited clinical/ echocardiographic detail. Single-centre retrospective series without robust adjustment for confounders^2,9^ and the non-randomized clinical trial^5^ were classified as high risk of bias, mainly owing to treatment-selection bias and lack of randomization or blinding.

The three secondary studies^6–8^ showed acceptable methodological transparency but inherited the limitations of the underlying observational data.

These constraints imply that our confidence is moderate regarding the *direction* of effect (mitral valve repair consistently appears associated with lower early mortality and fewer thromboembolic events, but with higher reoperation rates compared with replacement) whereas the magnitude of these effects remains uncertain. A sensitivity analysis excluding all high-risk studies^2,5,9^ did not materially alter the qualitative conclusions, although the total sample size and geographic diversity were reduced; therefore, the findings should be interpreted with appropriate caution.

The secondary studies^6–8^, were qualitatively appraised but not scored with NOS or RoB 2.

## 4. Discussion

This systematic review synthesizes evidence on long-term outcomes of mitral valve repair (MVP) versus replacement (MVR) in rheumatic disease. Overall, MVP was associated with better early safety and functional outcomes, whereas MVR offered more durable valve function in selected contexts. Surgical decisions must therefore balance these trade-offs in light of patient anatomy and severity. Below we discuss the main findings by outcome domain, highlighting key factors such as lesion subtype, baseline severity, and surgeon experience.

### 4.1. Early and late mortality

Early (30-day) mortality was significantly lower after MVP. In our pooled data, repair patients had an early death rate of only ∼0.96% versus 2.2% for replacement, consistent with prior studies^11^. Several mechanisms may explain this advantage. For example, MVP typically requires shorter cardiopulmonary bypass (CPB) time^12^ and causes less inflammatory stress^13^, which may translate into better immediate survival. (Indeed, emerging approaches like cerium-oxide nanocatalysts have even been proposed to mitigate oxidative injury)^14^. These perioperative benefits appear to persist: large meta-analyses^7^ and cohort studies have found markedly higher long-term survival among repair patients.

However, the survival benefit of repair is not universal. In cohorts of very complex rheumatic cases, mortality differences disappeared. For instance, there are studies^1^ that find no significant difference in early death when MVP was attempted in patients with heavily calcified valves and concomitant surgeries^15^. Similarly, when mitral surgery is combined with aortic valve replacement or multivalve operations, MVR may be preferable due to the technical complexity^16^. Finally, patient and system factors matter: in regions with late presentation and advanced rheumatic disease, established pulmonary hypertension and ventricular dysfunction drive mortality regardless of procedure^19^. Thus, while MVP generally confers lower mortality, this is moderated by baseline disease severity and operative context. In practice, patients selected for MVR often have more severe lesions (e.g., calcification or predominant stenosis), which also contributes to the observed outcome gap^15^. These findings underscore the need to individualize the choice of MVP vs. MVR based on valve anatomy and surgical setting.

### 4.2. Reoperations and durability

The chief limitation of MVP in rheumatic disease was its higher reoperation rate^20^. Across studies, 7–19% of repair patients required repeat mitral surgery, compared to < 2% after replacement. In our review, 364 of 429 total reoperations occurred in the MVP group. This vulnerability is supported by the literature: one study reported that repair carried a 4.3-fold higher reoperation risk than replacement^1^, and another found 5-year redo rates of 13.8% for MVP versus 4.2% for MVR^6^. These differences are attributed to the relentless rheumatic process: chronic inflammation causes recurrent leaflet fusion or degeneration even after an initially successful repair, whereas a prosthesis essentially “resets” the diseased valve^21^. For example, many reoperations in MVP patients were due to recurrent stenosis or regurgitation from ongoing disease. Patients with evidence of active rheumatic activity (e.g., high antistreptolysin-O titers) were particularly prone to repair failure^23^.

In subgroup terms, lesion type influenced durability. Isolated regurgitant lesions with pliable leaflets enjoyed better long-term freedom from reoperation, whereas patients with mixed or stenotic pathology had more frequent repair failures (often requiring MVR later). This pattern aligns with guideline recommendations: repairs are favored in dominant regurgitation, while MVR is indicated for predominant stenosis or diffuse calcification. In short, MVP offers less invasive early benefit but at the cost of more frequent late valve deterioration in rheumatic valves^24^.

Furthermore, the quality of the plasty depends directly on the technique used, and the variability in the results of the studies analyzed can be partially attributed to differences in surgical approaches. While some studies report the systematic use of a rigid ring with remodeling and leaflet resection, others do not specify technical standardization. The lack of uniformity makes more robust comparisons between the series difficult. Recent improvements in cardiac imaging have refined the preoperative evaluation of mitral valve anatomy, allowing a more reliable prediction of reparability^41,43^.

Technical advances have been described to mitigate early structural failure of the PM in VRM. It was introduced the “modified commissural release” technique, which promotes greater leaflet mobility in patients with Carpentier type IIIa restriction^5^. This approach showed a reduction in transmitral pressures and a lower residual gradient. It was reported a significant drop in reoperation rates, from 17% between 2000-2010 to 8% between 2011 and 2020 (*p* = 0.014), showing the positive impact of surgical evolution^2^. Also noteworthy is the four-stage protocol, described in one study, which combines commissural release, fibrous tissue removal, chordae tendineae shortening and annuloplasty with pericardial reinforcement^9^. This approach resulted in a reduction in the transvalvular gradient and a significant reduction in the diameter of the left atrium. Parallel advances in intraoperative assessment have further contributed to safer postoperative recovery and long-term surveillance, with early recognition of repair-limiting features^42,44^. It is also reported success in extensive reconstructions with resection of annulus calcifications and reinforcement with autologous pericardium, even in patients with hostile anatomy^25^.

Complementing this evidence, in a randomized clinical trial with 150 patients, it was demonstrated that a modification to the commissural release technique was able to reduce not only cardiopulmonary bypass time, but also peak diastolic velocity (1.2 ± 0.3 m/s compared to 1.6 ± 0.4 m/s; *p* < 0.01), which translated into less valve degeneration in the postoperative period, because it indicates a more efficient transvalvular flow during diastole, with less resistance and a lower pressure gradient^5^. This results in less mechanical stress on the valve leaflets and reduces turbulence^26^, factors which contribute significantly to avoiding microlesions and structural degeneration of the valve in the postoperative period.

When repair fails, the decision between conventional surgical reoperation or a transcatheter approach must be carefully weighed up. A study evaluated the results of the Valve in-Ring (ViR) technique in 128 patients with mitral valve repair failure. The technical success rate was 81%, with low in-hospital mortality (2.4%) and a significant reduction in transmitral gradients at 6 months^27^. This technique represents a promising alternative, especially in patients at high surgical risk or with comorbidities.

The transcatheter approach has also been gaining prominence with the introduction of mitral transcatheter edge-to-edge repair (mTEER) technology, especially with devices such as MitraClip™. Although traditionally indicated for degenerative mitral insufficiency, a study have shown that patients with mild to moderate residual rheumatic insufficiency can benefit from percutaneous intervention, delaying the need for open reoperation^17^. In parallel, in elderly patients with degenerative disease, conventional surgery still provides superior long-term survival, despite carrying a higher early postoperative risk^18^.

Finally, the international literature highlights the importance of accurately selecting patients who are candidates for repair. Factors such as young age, mobile leaflets, absence of extensive calcification and pure mitral regurgitation are strongly associated with the long-term success of MP. On the other hand, patients with predominant stenosis, anterior leaflet retraction, multivalvular involvement or the need for concomitant valve replacement should be considered for MVT, as reinforced by international guidelines such as those of the ESC (2021) and AHA/ACC (2020).

### 4.3. Thromboembolic and other complications

MVR, especially with mechanical valves, carried substantially more thromboembolic and bleeding complications. In the pooled studies, roughly three times as many thromboembolic events occurred after replacement as after repair, reflecting mandatory long-term anticoagulation with prostheses. For example, it was reported a 10-year thromboembolism rate of 7.6% in mechanical MVR patients versus 2.1% in the MVP group (HR ≈ 3.7)^1^. Correspondingly, hemorrhagic stroke and major bleeding were far more common after MVR: warfarin therapy in prosthetic patients was associated with intracerebral hemorrhage in ∼3.4% versus 0.9% of repair patients^28^.

By contrast, most MVP patients do not require chronic anticoagulation (unless they have atrial fibrillation or other indications). Recent evidence suggests that in bioprosthetic cases, antiplatelet therapy may suffice: aspirin alone had significantly lower bleeding than warfarin without increasing thrombosis risk^29,30^. This reinforces the safety profile of repair (which usually avoids warfarin altogether). In practice, subtherapeutic anticoagulation was common in many series: for instance, only 42% of MVR patients maintained target INR in one study, highlighting the challenge of strict anticoagulation management. Bioprosthetic valves present an intermediate picture: Ricciardi et al. (2024) reported that bioprostheses had a lower bleeding risk in the medium term compared to mechanical valves, though at the expense of eventual structural degeneration^31,32^.

Infective endocarditis was also more frequent after replacement. Historically, prosthetic material has higher infection risk than native tissue^33,34^. In our review, MVR patients had about a 2.5-fold higher endocarditis rate than MVP patients (OR ≈2.5 in one series). Such risk is particularly high in patients with a history of atrial fibrillation, uncontrolled systemic hypertension or advanced age, where anticoagulation is an independent risk factor for clinical complications^35^. Although PM reduces long-term anticoagulation needs^36^, it remains susceptible to recurrent mitral regurgitation, often requiring reoperation, especially in rheumatic valves^37^, demanding ongoing preventive care for thromboembolic and infectious complications.

Finally, pregnancy considerations strongly tilt toward MVP: it was noted that avoiding warfarin greatly benefits young women, since vitamin K antagonists are teratogenic and require frequent INR checks^40^. In summary, MVP’s avoidance of chronic anticoagulation confers major safety and quality-of-life advantages in thromboembolic and bleeding outcomes^28,29^, whereas MVR’s complications heavily reflect anticoagulation management.

### 4.4. Functional, hemodynamic and quality-of-life outcomes

#### 4.4.1. Ventricular function and hemodynamic performance

Comparing mitral valve repair (MVP) and mitral valve replacement (MVR) from the perspective of quality of life and ventricular function reveals essential nuances for surgical management, especially when considering the long-term clinical impact. Evidence suggests that MP provides superior functional and subjective advantages^8^: was identified a higher proportion of patients in NYHA functional class I-II after repair (89%) compared to those who underwent valve replacement (79%; *p* = 0.0012). This result reflects the more effective preservation of cardiac performance over time. This preservation is mainly related to the integrity of the subvalvular apparatus, maintained in the repair, and the lesser interference in ventricular dynamics promoted by the native valve^45^.

In the nine articles analyzed, mitral valve repair (MP) showed superior hemodynamic performance compared to MVT. The mean transvalvular gradient was lower (2.5 to 2.9 mmHg) compared to patients undergoing valve replacement (3.5 to 4.2 mmHg). These values indicate less obstruction to the left ventricular outflow tract and better cardiac output capacity during exercise, directly reflected in less fatigue and dyspnea on exertion. There is evidence that structural variations such as mitral annulus disjunction, particularly when associated with systolic curling, can generate localized mechanical stretch that contributes to regional ventricular remodeling in arrhythmic phenotypes^22^.

A study showed that the left ventricular ejection fraction was statistically higher in the PM group (60.7%) than in the MVT group (57.1%), indicating preservation of ventricular function^8^. This difference is attributed to the maintenance of the subvalvar apparatus during plasty, which preserves ventricular geometry and prevents pathological remodeling. In addition, the integrity of the papillary muscle and chordae tendineae guarantees synchronous movement of the valve with the ventricular myocardium, which is not the case with prosthesis replacement. Functional assessment using the NYHA criteria also favors PM. It was reported that 89% of patients undergoing PM were in functional class I or II after 12 months, compared to 79% in the MVT group^8^. This data is clinically relevant, as it indicates less physical limitation and greater autonomy in the post-operative period.

#### 4.4.2. Quality of life outcomes

Evaluations using the SF-36 and the Minnesota Living with Heart Failure Questionnaire show better quality of life after PM, especially in the domains of physical limitation, vitality and pain. In line with these findings, it was reported a faster return to activity and less use of diuretics in the PM group^5^. However, these gains depend on technical execution and clinical follow-up. This is also consistent with the general behavior of complex receptor-regulated systems, in which functional benefits are maintained only when key structural determinants remain stable over time^38^. Recurrence of mitral regurgitation can bring back symptoms such as dyspnea and effort intolerance, requiring reintervention and worsening quality of life^39^.

The absence of chronic anticoagulation in MP also improves quality of life, especially in young people and pregnant women. It was pointed out that frequent INR control impairs routine, increases anxiety and reduces adherence, especially in contexts with limited access to healthcare^31,40^. In addition, PM provides more freedom for physical activity and pregnancy planning, with a positive impact on the psychosocial sphere and professional reintegration^40^.

### 4.5. Clinical considerations and subgroup analyses

The choice of MVP versus MVR must integrate valve pathology, patient factors, and surgical expertise. In line with current guidelines, all the included studies stressed that favorable anatomy is key for repair success: patients with dominant regurgitation, mobile leaflets, limited calcification and no advanced stenosis fared best with MVP. Conversely, those with rigid, fibrotic valves or need for multiple valve interventions usually underwent replacement. International guidelines (ESC/EACTS 2021, AHA/ACC 2020) reflect these findings, strongly recommending repair in anatomically suitable rheumatic valves, but only when performed by experienced surgeons in high-volume centers. In practice, surgeon skill and institutional volume matter: complex rheumatic repairs require advanced techniques, so outcomes are generally better in specialized centers with extensive mitral expertise.

Theres are also important baseline differences between groups. Patients selected for MVR often had more severe disease at baseline (e.g., extensive calcifications, predominant stenosis), whereas MVP groups tended to have milder pathology. This selection bias likely contributes to some outcome differences: poorer early outcomes in the MVR cohort may partly reflect higher preoperative risk.

In summary, MVP is preferable when technically feasible and in patients with less severe, regurgitant-dominant disease^45^. The review’s overarching finding is that repair yields lower perioperative mortality, fewer long-term anticoagulant complications, and better functional recovery, while replacement provides more definitive relief of stenosis and valve durability. Surgical decisions should therefore be individualized, matching each patient’s lesion subtype (stenosis vs. regurgitation vs. mixed) and clinical profile to the optimal procedure, and carried out in settings of appropriate surgical experience. This evidence supports a tailored approach: select MVP for isolated regurgitant lesions in experienced hands, and reserve MVR for extensive stenotic or complex valves, recognizing the trade-offs in each scenario^15,45^.

## 5. Conclusion

This systematic review showed that mitral valve repair (MVP) has significant benefits in the treatment of rheumatic mitral valve disease compared to mitral valve replacement (MVR), especially in young patients with favorable valve anatomy.

There was a reduction in patient mortality (0.96% vs. 2.2%), improved hemodynamic performance (mean transvalvular gradient of 2.5 to 2.9 mmHg vs. 3.5 to 4.2 mmHg), more preserved ventricular function (left ventricular ejection fraction of 60.7% vs. 57.1%), lower incidence of thromboembolic events (114 vs. 323) and hemorrhagic events (39 vs. 252) in patients undergoing mitral valve repair. These findings, coupled with the lack of need for chronic anticoagulation and a 20-minute shorter cardiopulmonary bypass time, contribute to enhanced functional capacity and quality of life for patients undergoing MP, with 89% of patients in NYHA class I-II after repair, compared with 79% of valve replacement patients.

Conversely, mitral valve repair is associated with higher reoperation rates (7 to 19% vs. < 2%), approximately 3 to 9 times higher than MVP, a difference mainly attributed to mitral stenosis recurrence and progression of rheumatic valve degeneration. Valve replacement, in contrast, is the most indicated procedure in cases of predominant stenosis, anterior leaflet retraction, multivalvular involvement, the need for concomitant valve replacement or extensive calcifications, despite the risks related to anticoagulation, prosthetic deterioration and thromboembolic events. Advanced repair techniques, such as commissureplasty and the use of autologous pericardium, have shown a reduction in reoperation rates (<6% at 5 years) and improved hemodynamic performance.

Although mitral valve repair is associated with lower early mortality rates, fewer thromboembolic and hemorrhagic events, and favorable long-term functional outcomes when anatomically feasible, rheumatic mitral valve disease does not demonstrate clinical equipoise due to higher reoperation rates with repair. The preferred strategy remains strongly context-dependent, conditioned by valve morphology, extent of calcification or fibrosis, patient age, anticoagulation feasibility, and surgical expertise, requiring individualized decisions within experienced multidisciplinary teams.

Nevertheless, there is still a need for more longitudinal studies and randomized clinical trials focused exclusively on rheumatic valvular heart disease, so that repair techniques can be standardized, long-term outcomes can be clarified and the equivalence of efficacy between approaches can be confirmed, thus ensuring more precise and individualized surgical decisions to optimize clinical results and patients’ quality of life.

## Author statement

**Conceptualization:** Sofia Araujo and Fernanda Chaves.

**Software:** Arthur de Lima.

**Writing –Original Draft Preparation:** Sofia Araujo, Renato Filho, Natalia Alves, Arthur de Lima and João Aires.

**Writing –Review & Editing:** Sofia Araujo, Natalia Alves, João Aires, Hugo Silva and Fernanda Chaves.
